# A Case Report on Biliary Ascariasis

**DOI:** 10.7759/cureus.33323

**Published:** 2023-01-03

**Authors:** Jouhar J Kolleri, Amal M. J. Thabet, Shahd Mohammedain, Sadia Sajid, Zahoor Ahmed, Umais Momin

**Affiliations:** 1 Clinical Imaging Department, Hamad Medical Corporation, Doha, QAT; 2 Radiology Department, Hamad General Hospital, Doha, QAT

**Keywords:** ercp, ultrasound imaging, ascariasis, biliary ascariasis, ascariasis lumbricoides

## Abstract

Biliary Ascariasis occurs when Ascaris lumbricoides worms invade the biliary system. It may cause biliary obstruction, cholangitis, cholecystitis, or acute pancreatitis. We report a case of a 37-year-old female patient who presented with a history of upper abdominal pain, nausea, vomiting, and weight loss for two weeks. Ultrasound showed dilated common bile duct with linear tubular echogenic structure in the common bile duct and bowel loops. Endoscopic Retrograde Cholangio pancreatography (ERCP) revealed large adult worms confirming the diagnosis of Ascariasis.

## Introduction

Ascariasis, a helminthic infection caused commonly by Ascaris lumbricoides, infects about 33% of the world’s population making it one of the most common gastrointestinal tract parasitic infestations [1]. Humans are infested by ingestion of ova-contaminated food and water. Gastric acid then stimulates the mature ova to hatch into larvae that migrate and invade various parts of the body including the cecum, liver, pancreas and lungs [1,2]. Most patients are asymptomatic; however, hepatobiliary disease can present with complications of biliary obstruction like biliary colic, acute cholangitis, acute cholecystitis, and acute pancreatitis, especially in those with a large worm load [3]. Other symptoms like anorexia, nausea, vomiting, diarrhea, bloating, abdominal distention, and discomfort are equally common [4]. Herein, we present a case of biliary Ascariasis that was successfully managed with Endoscopic Retrograde Cholangio pancreatography (ERCP), stent placement, intravenous fluids, and albendazole for deworming.

## Case presentation

A 37-year-old lady with no chronic medical illness presented with two weeks history of episodes of upper abdominal pain. The pain was dull in nature, non-radiating, and worsening with movements and change in position, which did not get relieved with antacid or analgesics. It started gradually without a specific pattern and sometimes reached 10/10 in severity. She also experienced anorexia for one month, nausea and vomiting for three days duration. There was a history of significant loss of weight of 5 kg over the last month. She recently came from the Philippines and working as a housemaid. The patient denied any history of diarrhea, constipation, hematemesis and melena.

**Figure 1:**
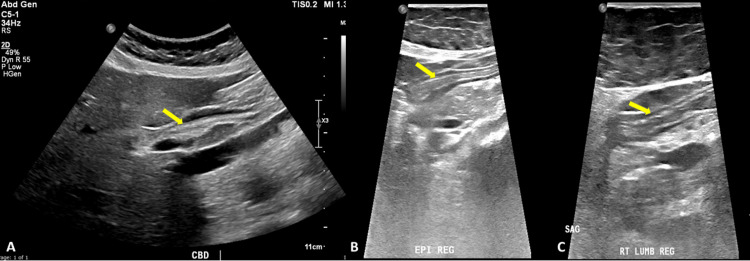
Ultrasound of abdomen showing a linear echogenic structure in the A) CBD, B) epigastric region and C) right lumbar area (yellow arrows).

Her vitals were within normal limits. On examination, the patient was conscious and oriented. The abdomen was soft, and lax, with severe epigastric tenderness. There was no palpable mass or hepatosplenomegaly. Ultrasound abdomen was done which showed dilated common bile duct (CBD), measuring 9 mm. There was a linear tubular echogenic structure noted in the common bile duct. There was another linear echogenic structure noted within the bowel loops in the epigastric region and the right lumbar area. Other major visualized abdominal organs were unremarkable. These linear echogenicities were likely due to ascariasis (Figures 1A-1C).

**Figure 2:**
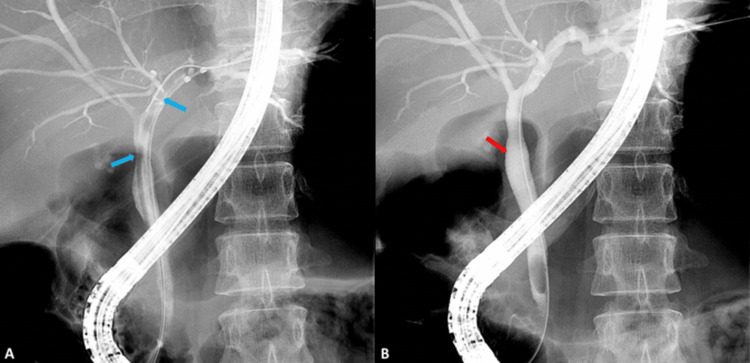
Cholangiogram showing A) a big adult worm in the left hepatic duct and proximal CBD (blue arrows) and B) absent adult worm after removal using guidewire (red arrow).

The gastroenterology team was consulted and advised ERCP. On ERCP, the stomach showed a large adult worm, which was stuck to the suction channel and removed out. The first and second parts of the duodenum showed multiple big adult worms of ascariasis. Cholangiogram showed one big adult worm in the left hepatic duct and proximal CBD. The guide wire was passed selectively to the left hepatic duct and the adult worm was removed with balloon weep (Figures 2A, 2B). CBD became normal, cleared off worms, and a 5 cm 8.5 Fr double pigtail stent was placed deep into the left hepatic duct.

The patient was given 400 mg albendazole tablet, weekly once for two weeks, intravenous fluid became symptomatically better, and advised to follow up in the GI clinic for stent removal after deworming.

## Discussion

Ascariasis infection, a global parasitic infection by the organism “Ascaris lumbricoides”, is most commonly distributed in tropical and subtropical areas. The most common cause of the spread of Ascariasis infection is poor hygienic precautions and poverty [5]. Humans are infested after ingestion of contaminated food, the eggs reach as larvae to the jejunum, where they hatch, then they enter the portal blood circulation to reach the liver after breaking through the intestinal wall followed by departing to the lungs. The larvae evolve and molt two times to be capable of penetrating the capillary membranes of the alveoli and ascend to the upper respiratory tract. They get back to the gut by swallowing in the form of adult worms [6].

The clinical presentation of Ascariasis infection is either symptomatic or asymptomatic with the vast majority of symptomatic infection occurring in case of a significant parasitic load. The symptoms develop according to the phase of the parasite’s life cycle; larvae in the lungs are manifested by pulmonary symptoms, while adult worms in the bowels are manifested clinically by non-specific abdominal symptoms, including abdominal pain, nausea, vomiting, anorexia and diarrhea, like our case. Adult worms’ transfer through the gastrointestinal tract may lead to complications, host present mostly with a complaint of an acute abdomen. When worms travel through the ampulla of vater to the biliary tract, pancreas, and liver, liver abscess, acute cholecystitis, and acute pancreatitis might occur [7]. The bulky intestinal parasitic load can form a bag of worms that can complicate bowel obstruction and intestinal hemorrhage because of perforated or ulcerated bowel walls [5,7].

The diagnosis of Ascariasis infection in developed countries cannot depend exclusively on the history, clinical features, and stool analysis. Many medical imaging methods have been well-evolved, such as ultrasonography, considered an initial and reliable diagnostic tool for biliary Ascariasis [8]. The worms in the biliary tract can appear on ultra-sonographic images in variable manifestations, including inner-tube sign and strip sign in gall bladder and CBD; the worm is shown as an echoic thick stripe with a longitudinal centralized non-echoic tube and as a thin linear and tubular echogenic stripe without an inner tube or shadowing, respectively. While in the main bile duct, it is seen as a linear echogenic interface, called a spaghetti sign. Intrahepatic duct Ascariasis is far less common than CBD’s, it appears on ultrasound images with a dilated intrahepatic duct, as a calcified structure, stripe sign, or triple line in high-resolution images. Intestinal Ascariasis appears as a linear echoic stripe with a non-echoic inner canal, four paralleled arranged lines with three separating bands, and a round structure of a bag of worms in case of multiple worms [9]. In our study, abdominal ultrasonography showed a dilated common bile duct, in association with a linear tubular echogenic structure. Another linear echogenic structure was noted within the bowel loops in the epigastric region and the right lumbar area.

Ultrasound is an easy, highly sensitive, strongly specific, safe, and non-invasive modality and it has been used as the first imaging tool for biliary Ascariasis diagnosis, especially when the worm is moving, the typical shape of it on ultrasound is a linear echoic with no shadowing structure. Three modalities are available for the treatment of Ascariasis: non-surgical management, surgical management for patients with poorly responsive to the medications or those who are complaining of severe infection, and endoscopic management. ERCP represents an endoscopic diagnostic and therapeutic method, which has been efficiently used to visualize and remove the worms from the biliary tract at the time of diagnosis [10].

Literature research reported about a Korean case of biliary Ascariasis infection, ERCP was used as a confirmatory diagnostic and therapeutic method, the worm was extracted through the ampulla of the vater by using the balloon sweeping method, the treatment was completed with the administration of medical management using the drug “albendazole”, the patient was discharged without any complication [11]. Our patient was also treated in the same way, got symptomatically better, and got discharged home.

## Conclusions

The chief complaints of Ascariasis infection are non-specific abdominal symptoms; as a result, it may be easily misdiagnosed with other diseases. Therefore, in endemic areas, the parasitic infection should be one of the differentials of an acute abdomen. Ultrasonography has achieved great success in confirming the diagnosis. Radiologists need to be aware of the radiologic features and manifestations of parasites. ERCP in association with anthelmintic drugs has been properly documented as the mainstay treatment modality.
